# Crystal Structure Defects in Titanium Nickelide after *Abc* Pressing at Lowered Temperature

**DOI:** 10.3390/ma15124298

**Published:** 2022-06-17

**Authors:** Aleksandr Lotkov, Victor Grishkov, Roman Laptev, Yuri Mironov, Dorzhima Zhapova, Natalia Girsova, Angelina Gusarenko, Elena Barmina, Olga Kashina

**Affiliations:** 1 Institute of Strength Physics and Materials Science of the Siberian Branch of the Russian Academy of Science, 634055 Tomsk, Russia; grish@ispms.ru (V.G.); myp@ispms.tsc.ru (Y.M.); dorzh@ispms.tsc.ru (D.Z.); girsova@ispms.tsc.ru (N.G.); aag@ispms.ru (A.G.); barmina@ispms.ru (E.B.); ocash@ispms.ru (O.K.); 2 Division for Experimental Physics, National Research Tomsk Polytechnic University, 634050 Tomsk, Russia; laptevrs@tpu.ru

**Keywords:** titanium nickelide, *abc* pressing, dislocation density, vacancies, positron annihilation spectroscopy

## Abstract

The experimental results regarding the effect of warm (573 K) *abc* pressing with an increase in the specified true strain, *e*, up to 9.55, on the microstructure and crystal structure defects (dislocations, vacancies) of the Ti_49.8_Ni_50.2_ (at %) alloy are presented. It is shown that all samples (regardless of *e*) have a two-level microstructure. The grains–subgrains of the submicrocrystalline scale level are in the volumes of large grains. The average sizes of both large grains and subgrain grains decrease with increasing *e* to 9.55 (from 27 to 12 µm and from 0.36 to 0.13 µm, respectively). All samples had a two-phase state (rhombohedral R and monoclinic B19′ martensitic phases) at 295 K. The full-profile analysis of X-ray reflections of the B2 phase obtained at 393 K shows that the dislocation density increases from 10^14^ m^−2^ to 10^15^ m^−2^ after pressing with *e* = 1.84 and reaches 2·10^15^ m^−2^ when *e* increases to 9.55. It has been established by positron annihilation lifetime spectroscopy that dislocations are the main type of defects in initial samples and the only type of defects in samples after *abc* pressing. The lifetime of positrons trapped by dislocations is 166 ps, and the intensity of this component increases from 83% in the initial samples to 99.4% after pressing with *e* = 9.55. The initial samples contain a component with a positron lifetime of 192 ps (intensity 16.4%), which corresponds to the presence of monovacancies in the nickel sublattice of the B2 phase (concentration ≈10^−5^). This component is absent in the positron lifetime spectra in the samples after pressing. The results of the analysis of the Doppler broadening spectroscopy correlate with the data obtained by the positron annihilation lifetime spectroscopy.

## 1. Introduction

Methods of severe plastic deformation (SPD), or megaplastic deformation, grind the grain–subgrain structure to a submicro- and even nanocrystalline state in metallic materials [1,2], which leads to a significant increase in the yield strength and strength while maintaining satisfactory plasticity. The TiNi-based alloys exhibit shape memory effects (SME) and superelasticity and belong to the class of intelligent materials that have found application in engineering and medicine [3]. The *abc* pressing [4,5] and equal channel angular pressing (ECAP) [1,6,7,8] are promising methods for obtaining massive semi-finished products from TiNi-based alloys with an ultrafine-grained (UFG) structure. The regularities and features of microstructure evolution in TiNi-based alloys under the action of SPD at 623–773 K are studied in a number of works: [9,10,11,12,13,14,15,16,17,18,19,20,21] after ECAP and [4,5,22,23] after *abc* pressing. The effect of grain–subgrain structure refinement on the mechanical and inelastic properties of these alloys after ECAP was studied in [9,10,11,12,13,14,15,16,17,18,19,20,21] and after *abc* pressing in [22,23,24,25,26]. It was found that these methods can achieve a refinement of the grain–subgrain structure up to 100–500 nm, depending on the pressing temperature. It has also been established that samples of these alloys with an average grain–subgrain structure of 250–300 nm demonstrate an ultimate tensile strength of up to 1200 MPa, and the plasticity reaches 50–60%. In [1,8,14,15,22,23,24,25,27], the effect of ECAP and *abc* pressing on the martensitic transformation (MT) temperatures in TiNi-based alloys was studied. The MT temperatures during the cooling and heating of samples of these alloys decrease by 20 degrees or more after ECAP at 623–773 K [1,8,14,15,27], and after *abc* pressing, they either do not change or change only slightly [22,23,24,25]. It is shown in [11] that the dislocation density in Ti_50_Ni_50_ and Ti_49.2_Ni_50.8_ alloys after ECAP equals 10^15^–5.3·10^15^ m^−2^. A higher dislocation density (10^17^–10^18^ m^−2^) was found in the transition region between crystalline and amorphous phases after cold rolling of Ti_49.2_Ni_50.8_ alloy [28]. At the same time, it should be noted that for the analysis and understanding of the mechanisms of grain structure refinement in alloys under the influence of SPD, there are not enough works on studying the evolution of both dislocations and other defects in the crystal structure. It is known that during SPD, not only dislocations are accumulated in the samples but vacancies also appear, the concentration of which can be much higher compared to the thermodynamic equilibrium one [29,30]. A high concentration of vacancies accelerates the mass transfer, dissolution, or segregation of secondary phases [31,32] and, similarly to dislocations, stimulates the formation of a new grain–subgrain structure, which increases the length of grain boundaries in samples [33]. It is also known that under conditions of high concentration and low mobility of vacancies, they can form clusters [34], which can lead to a decrease in the long-term strength of materials [35,36]. Therefore, the study of the formation of free volumes in the form of vacancies in metallic materials subjected to SPD is an urgent task, since its solution will contribute to a better understanding of the deformation behavior of UFG materials. The study of vacancies generated during SPD [29,37,38,39,40,41] was carried out mainly at room temperature, when their mobility is low for most metals. To study vacancies and their clusters, as well as the density of dislocations in metals and disordered solid solutions after SPD, various physical methods are used (electrical resistance [30], dilatometry [38], differential scanning calorimetry [30,38], X-ray line profile analysis [42]), as well as methods of perturbed angular correlation of gamma rays (PAC) [43] and positron annihilation spectroscopy (PAS) [37,38,39,40,41]. The results of studies by these methods show comparable values of the relative concentrations of vacancies in pure metals and disordered solid solutions after SPD: 10^−2^–10^−4^. At the same time, there are very few data on vacancies that appear after SPD in intermetallic compounds. The vacancy defects in these compounds are more diverse than in pure metals and disordered solid solutions [44]. For example, in Fe_3_Si samples, which have a nanocrystalline structure after SPD in ball mills, vacancy-like defects were detected by the PAS method, which can be attributed to interface defects [45]. In [46], a comparison of the experimental results obtained by differential scanning calorimetry with model calculations showed that high-pressure torsion of FeAl intermetallic samples with a B2 structure can achieve a vacancy concentration of 10^−2^. In [47], using the PAC method, it was found that Schottky pairs are predominant defects that form during the grinding of PdIn samples in a ball mill, triple defects in NiAl and FeRh samples, and antistructural defects in FeAl. It was shown in [48,49] that the relative concentration of monovacancies in the surface layers of the TiNi intermetallic compound of equiatomic composition after ultrasonic impact treatment at room temperature increases to ~10^−5^.

The purpose of this work is to present the results of a comprehensive study of the effect of *abc* pressing at 573 K on the types of formed defects in the crystal structure, their evolution and density depending on the value of true strain in samples of T_49.2_Ni_50.2_ (at %) alloys.

## 2. Materials and Methods

The binary alloy Ti_49.8_Ni_50.2_ (at %) was chosen for research. This alloy has higher ductility than the binary alloys with the higher Ni content. This made it possible to set large deformations for the samples at a low *abc* pressing temperature (573 K). The alloy was received by MATEK-SMA Ltd. (Moscow, Russia) in the form of bars (ø20 mm). Samples in the form of a cube with dimensions of 20 × 20 × 20 mm^3^ were obtained by pressing blanks (ø20 mm, length 25 mm) at 1073 K. The cyclic warm deformation of the samples was carried out at (573 ± 10) K (0.36 T_m_, where T_m_ is the melting temperature). The rate of deformation in one act of compression was 0.15–0.18 s^−1^. In each cycle of deformation, the sample was subjected to compression in three mutually perpendicular directions. The cycles were repeated until the required specified true strain was reached. After *abc* pressing, samples were obtained with given values of true strain *e* equal to 1.84, 3.60, 5.40, 7.43, and 9.55.

The microstructure and phase composition of samples after *abc* pressing was studied on the equipment of the Shared Use Center “NANOTECH” by transmission electron microscopy (TEM) JEM-2100 (JEOL Ltd., Tokyo, Japan). Foils for TEM are prepared either by electrolytic polishing in an electrolyte containing sulfuric, nitric and hydrofluoric acids in a ratio of 6:1:3 or by ion etching on an Ion-slicer EM-09 100 15 device (JEOL Ltd., Tokyo, Japan). The average sizes of grains and subgrains, ⟨d⟩, were determined by measuring the average diameter of no less 100 grains on bright- and dark-field TEM images of sample microstructures.

The microstructure of the samples was also studied by optical microscopy using an AXIOVERT-200MAT (Carl Zeiss AG, Oberkochen, Germany). The average size of coarse grains, ⟨D⟩, was determined from differential interference contrast (DIC) images of sample microstructures. Details of the grain structure of the samples (in particular, grain boundaries) are more clearly expressed in DIC images than in traditional bright-field optical images. The average grain size, ⟨D⟩, was calculated using the formula:

(1) $$\langle D\rangle ={\left(S/N\right)}^{1/2},$$

where *S* is the area of DIC images of the microstructure containing *N* grains (*N* is about 120).

Samples for studies by X-ray diffraction analysis (XRD) and optical microscopy were prepared in the form of plates with dimensions of 11 × 18 × 2 mm^3^. The surfaces of the samples were mechanically ground using a Saphir 350 grinder and polisher (Audit Diagnostics, Business & Technology Park, Carrigtwohill. Co., Cork, Ireland) using a silicon carbide abrasive paper with a gradual decrease in grain size to 1200. Finally, the surface of the samples was brought to a diamond paste with a dispersion of 3 microns. To remove the work-hardened layer, the surface of the mechanically polished samples was chemically etched in an etchant of the following composition: nitric acid, hydrofluoric acid and water (hydrofluoric acid, nitric acid and water) in a ratio of 1:4:5. The same samples were used to study the defects in the crystal structure of samples after *abc* pressing by the positron lifetime spectrometry and the coincidence of the Doppler broadening of the annihilation line.

The scalar density of dislocations in the samples was determined by XRD. Diffractograms from samples with the structure of the cubic B2 phase were obtained at a temperature of 393 K (above the temperature A_f_ of the end of the MT when the samples were heated) using an HTK 2000N camera (Anton Paar, Graz, Austria) and XRD-7000S diffractometer (Shimadzu, Kyoto, Japan) with filtered Cu-K_α_ radiation and a high-speed linear 1280-channel detector OneSight FD-1001 (Shimadzu) [50]. The diffraction patterns were processed using the PDWin software package (JSC IS Bourevestnik, St. Petersburg, Russia). The Williamson–Hall full-profile analysis method [51,52,53] was used to separate the contributions to the physical broadening of the X-ray reflections from the magnitudes of microstrains and the average size of coherent scattering regions (CSRs). The next formula was used for the estimation of dislocation density [54,55]:

(2) $$\rho_{d}=2\sqrt{3}\frac{{\langle \varepsilon }^{2}\rangle {}^{1/2}}{\left\langle D_{CSR}\right\rangle b},$$

where ${\langle \varepsilon }^{2}\rangle$^1/2^ are the root-mean-square microstrains of the B2 phase crystal lattice, $\left\langle D_{CSR}\right\rangle$ is the size of *CSR*s, and *b* is the Burgers vector of dislocations.

To analyze structural defects by positron spectroscopy, we used a hybrid digital positron spectroscopy complex with an external synchronization system based on positron annihilation lifetime spectrometry (PALS) modules and coincidences Doppler broadening spectroscopy (CDBS) [56]. The time resolution of the PALS module is 172 ± 6 ps, and the count rate is 110 ± 30 counts/s. The count rate of positron–electron annihilation events for the CDBS module is 130 ± 20 counts/s with an energy resolution of 1.15 ± 0.04 keV. The ^44^Ti isotope with an activity of 1.38 MBq and a maximum positron energy of 1.47 MeV was used as a source of positrons. 

For each sample, four positron lifetime spectra and one two-dimensional CDBS spectrum were collected with statistics of 3·10^6^ and 2·10^7^ annihilation events, respectively. The positron lifetime spectra were processed using the LT10 software (version 10.2.2.2, University of Silesia, Katowice, Poland) [57,58] according to the three-component positron trapping model, which takes into account the annihilation of delocalized positrons in the lattice as well as the annihilation of positrons trapped by two types of defects [59]. Three components of the positron lifetime were used for the analysis: τ_A_ (positron capture by type A defects), τ_B_ (positron capture by type B defects), τ_F_ (delocalized positron annihilation) and their relative intensities I_A_, I_B_, I_F_ = (100%-I_A_–I_B_). The experimental spectra were approximated by a series in which the variables τ_A_, τ_B_ were combined into a system for four lifetime spectra obtained for each sample, and the τ_F_ component was combined for all spectra. In this case, the lifetimes of positrons trapped by type A and B defects, as well as the lifetime of delocalized positrons in the TiNi lattice, are determined with high accuracy. The contribution of the positron source (≈9%) was corrected using the empirical function that was determined earlier and is characteristic of this source and the spectrometric module. The positron annihilation components in the source were τ_1_ = 142 ± 1 ps (65.3%), τ_2_ = 413 ± 1 ps (22.8%), τ_3_ = 1223 ± 10 ps (11.9%). 

Two-dimensional CDBS spectra were processed using the CDBTools software (Slovak University of Technology, Bratislava, Slovakia) [60]. To analyze the spectra, we used the traditional S and W DBS shape parameters [59] obtained for the cross-section along the abscissa of a two-dimensional spectrum. The parameter S is defined as the ratio of the number of positron annihilation events (i.e., area) in the central part of the annihilation peak (in the range from 490 to 520 keV) to the total number of positron annihilation events. This parameter characterizes the probability of annihilation of positrons with free electrons (low momentum). The W parameter is defined as the ratio of the number of positron annihilation events in the region of the Gaussian wings of the annihilation peak to the total area of the positron spectrum. This parameter characterizes the probability of annihilation of positrons with half-core and core electrons (high momentum values), and it is sensitive to changes in the chemical environment at the annihilation site. In addition, the relative dependences R(p) = N(p)/N_0_(p) were also analyzed for the cross-section along the abscissa axis of the two-dimensional spectrum, where N(p) is the annihilation photon spectrum of the sample under study and N_0_(p) is the reference spectrum. The spectrum of annihilation photons of a defect-free aluminum single crystal was used as the reference spectrum N_0_(p).

## 3. Results

**Figure 1:**
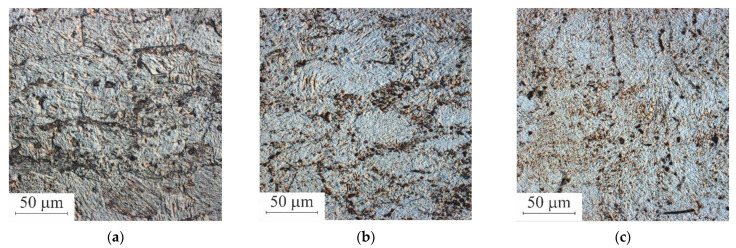
Microstructure of Ti_49.8_Ni_50.2_ alloy samples in the initial state (**a**) and after *abc* pressing with *e* = 3.60 (**b**) and *e* = 7.43 (**c**) Optical microscopy (DIC).

Images of the microstructure of Ti_49.8_Ni_50.2_ alloy samples in the initial state and after *abc* pressing at 573 K, obtained by optical microscopy (differential interference contrast-DIC), are shown in Figure 1. In the initial state, Figure 1a, the samples had a coarse-grained structure with an average grain size ⟨D⟩ ≈ 27 µm. Inside these grains, a developed structure of microbands is observed, which is the result of high-temperature pre-forming of cube-shaped samples. After *abc* pressing to the true strain *e* = 3.60, Figure 1b, and *e* = 5.40, the structure of microbands inside the grains of the samples is preserved. The average grain size ⟨D⟩ decreases with increasing *e* and becomes equal to ⟨D⟩ ≈ 15 µm after pressing with *e* = 5.40. After pressing with *e* = 7.43, Figure 1c, and *e* = 9.55, the microstructures of the samples are qualitatively similar and significantly more uniform than in the previous stages of pressing. In these samples, the presence of large grains is still observed, but their average size has become less than ≈12 µm.

The results of studies of the structural-phase composition of samples after *abc* pressing by transmission electron microscopy (TEM) confirmed the presence of a fine structure in the form of microbands inside large grains and showed that regardless of the value of the true strain specified during *abc* pressing, only martensitic phases with rhombohedral R and monoclinic B19′ structures are observed. In addition, a finer grain–subgrain structure is observed inside the grains of both the initial samples and the samples after pressing. The average value of these grains–subgrains ⟨d⟩, which is determined from the dark-field images of the microstructure of the samples, depending on the specified true *abc* strain, is shown in Table 1. From Table 1, it can be seen that with an increase in the true strain of the samples, *e*, the average value of these grains–subgrains decreases by a factor of ≈3, and it is equal to 0.13 μm at *e* = 9.55.

**Figure 2:**
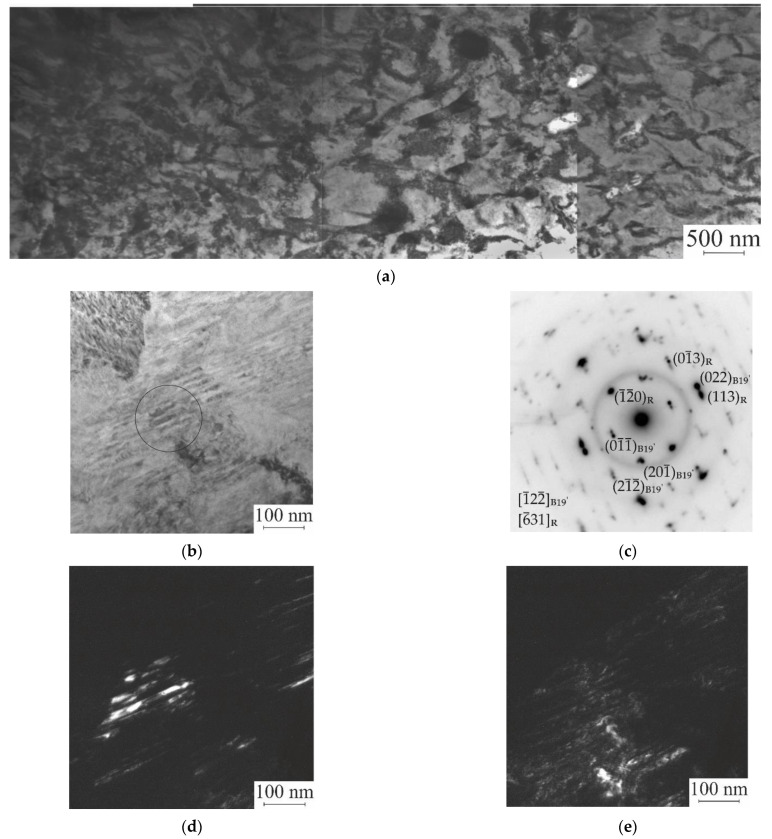
Images (TEM) of the microstructure of samples in a bright field after *abc* pressing up to *e* = 7.43: panorama of the area 4.5 × 9 μm^2^ (**a**) and the area at a distance of 255 μm from it (**b**) microdiffraction pattern (**c**) from a region with a two-phase R + B19′ structure, indicated by a circle in Figure 2b images in the dark field: in the reflection (022) of the B19′- phase (**d**) and in the reflection (113) of the R phase (**e**).

In general, after pressing to *e* = 7.43 and *e* = 9.55, the microstructure inside most grains becomes qualitatively similar and homogeneous. This is clearly seen in Figure 2a, which shows a bright field image of a large area (4 μm × 9 μm) of a sample with a given strain *e* = 7.43. The microstructure of this region is based on grains–subgrains, the maximum size of which does not exceed 500 nm. An enlarged image of a microdomain in a bright field located at a distance of ≈255 μm from the area shown in Figure 2a is presented in Figure 2b. Fragments of the grain–subgrain structure, the minimum size of which is ≈200 nm, are visible. Inside such fragments, regular structures of microbands (width of 30–40 nm) are observed. Analysis of the microdiffraction pattern, as shown in Figure 2c, obtained from the area highlighted in Figure 2b, showed that this area has a two-phase structure, including the rhombohedral R and monoclinic B19′ phases. Images of fragments with structures of R and B19′ phases in the dark field are shown in Figure 2d,e. In addition, the composition of the microstructure shown in Figure 2a contains both grains–subgrains with a two-phase structure R + B19′ and grains–subgrains in which either a martensitic R-phase or a martensitic B19′-phase are observed. The more detailed analysis of phase state and microstructure in Ti_49.8_Ni_50.2_ alloy after *abc* pressing at 573 K are presented in [61].

It is natural to expect that the formation of a submicrocrystalline structure inside the large grains and, accordingly, a decrease in the size of grains–subgrains and an increase in the length of their boundaries is due to a significant increase in the dislocation density in the samples with an increase in the given true strain specified during *abc* pressing at 573 K.

**Figure 3:**
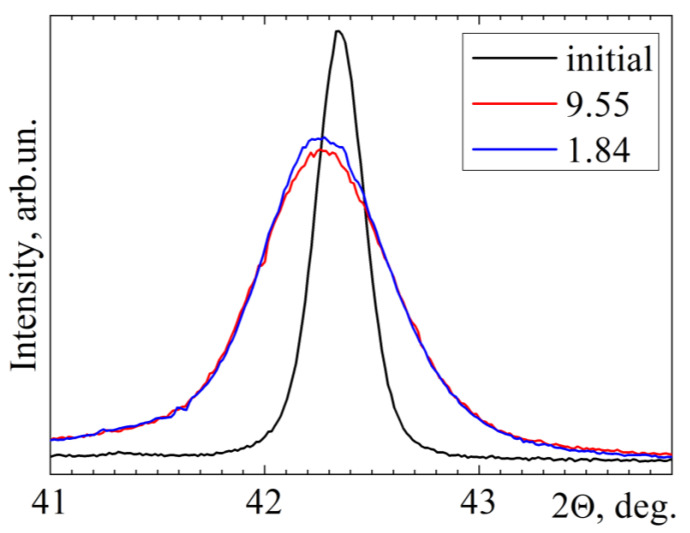
(110)_B2_ X-ray reflection profiles for Ti_49.8_Ni_50.2_ samples at a temperature of 393 K in the initial state and after *abc* pressing to *e* = 1.84 and *e* = 9.55.

Figure 3 shows the X-ray reflection profiles of (110)_B2_ from samples with true strains *e* = 1.84 and *e* = 9.55 and the reflection profile from the initial sample. It can be seen that the most significant changes are observed after *abc* pressing with *e* = 1.84. These changes consist in increasing the integral width of the reflection profile and shifting its position toward smaller diffraction angles. A subsequent increase in strain from 1.84 to 9.55 has little effect on the position and half-width of the (110)_B2_ reflection. Changes in the profiles of reflections (211)_B2_ and (220)_B2_ are qualitatively similar. The reflections with higher indices in the samples already after *abc* pressing with *e* = 1.84 have very low intensities and therefore were not analyzed.

The unit cell parameter of the B2 phase increases from 3.0182 ± 0.0003 Å in the initial samples to 3.021 ± 0.001 Å in all deformed samples. An increase in the half-width of reflections for samples after *abc* pressing indicates an increase in the average value of microstrains, ⟨*ε^2^*⟩^1/2^, of the crystal lattice of the B2 phase and a decrease in the average size of coherent scattering regions (CSRs), $\left\langle D_{CSR}\right\rangle$. The average value of microstrains increases from 0.2% in the initial samples to 0.8% in the samples after pressing with *e* = 1.84 and slightly decreases (to 0.7%) as *e* increases to 9.55. The CSRs sizes in the initial samples are 250 nm. After *abc* pressing with *e* from 1.84 to 9.55, $\left\langle D_{CSR}\right\rangle$ linearly decreases from 100 to 37 nm. In general, $\left\langle D_{CSR}\right\rangle$ is only 1.4–3 times smaller than the average grain–subgrain sizes determined by TEM in these samples, as shown in Table 1. 

**Figure 4:**
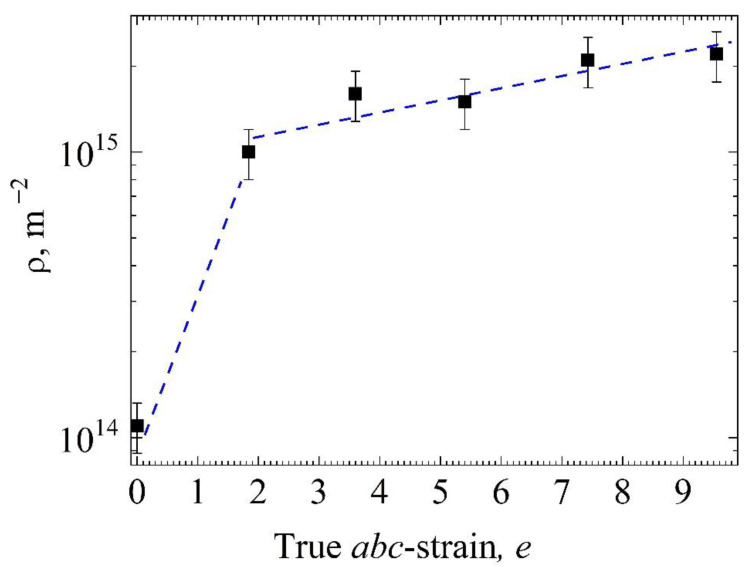
The scalar dislocation density as a function of the value of the true *abc* strain *e*.

The value of the scalar dislocation density depending on the *abc* strain specified for the samples is shown in Figure 4. The dislocation density in the samples after pressing with *e* =1.84 increases by an order of magnitude compared to the initial state (from 0.1·10^15^ m^−2^ to 1·10^15^ m^−2^). A subsequent increase in the *abc* strain to *e* = 9.55 leads to an increase in the dislocation density only by a factor of 2.

The XRD data correlate with the results obtained by positron annihilation lifetime spectroscopy. The parameters of the components obtained from the experimental positron lifetime spectra for Ti_49.8_Ni_50.2_ samples are presented in Table 2.

The spectrum component with τ_F_ = 138 ± 1 ps is close to the lifetime of delocalized positrons in the B2 phase of TiNi-based alloys. The experimental values of τ_F_ determined in previous works are: 132 ps [62], 140 ps [63], 128 ps [64]. The theoretical values of τ_F_ are 120 ps [65] and 127 ps [64]. The positron annihilation spectrum component τ_F_ is observed both in the initial samples and in the samples after *abc* pressing, but its intensity is very low (≤0.6%), as shown in Table 2. In this case, saturated positron trapping is observed in all samples. In the initial samples, the experimental spectrum of the positron lifetime contains two intensive components: τ_A_ = 169 ± 1 ps and τ_B_ = 192 ± 2 ps, as shown in Table 2. A similar component with a close value τ_A_ = 166 ± 1 ps is observed in all samples after *abc* pressing with specified strains from 1.84 to 9.55. These values are close to the lifetimes τ_A_ 159 ± 3 ps and 160 ps associated with the annihilation of positrons trapped by dislocations and experimentally observed in TiNi-based alloys in [63] and [64], respectively. The intensity of the I_A_ component in the initial samples is 83.4%, increases to 99.9% after pressing with *e* = 1.84, and remains almost unchanged as *e* increases to 9.55. 

Table 2 shows that the initial samples contain a long-lived component with τ_B_ = 192 ± 2 ps. The positron lifetime 192 ± 2 ps is close to the experimental value 197 ± 2 ps (the lifetime of positrons trapped by vacancies) [62], and the theoretical lifetimes of positrons trapped by vacancies on the sublattices of titanium $\tau_{V_{Ti}}$ and nickel $\tau_{V_{Ni}}$ atoms in various phases of titanium nickelide were calculated in [64]. It was shown in [64] that $\tau_{V_{Ni}}$ have similar values both in the B2 phase (194 ps) and in the R (199 ps) and B19′ (195 ps) martensitic phases, while $\tau_{V_{Ti}}$ = 207 ps for all these phases. Therefore, it is most likely that the τ_B_ component corresponds to the annihilation of positrons trapped by vacancies in the nickel sublattice of the Ti_49.8_Ni_50.2_ alloy. This component is absent in the lifetime spectra of samples after *abc* pressing with *e* from 1.84 to 9.55. Thus, the main type of defects in the initial samples and the only type of defects in the samples after *abc* pressing are dislocations. 

**Figure 5:**
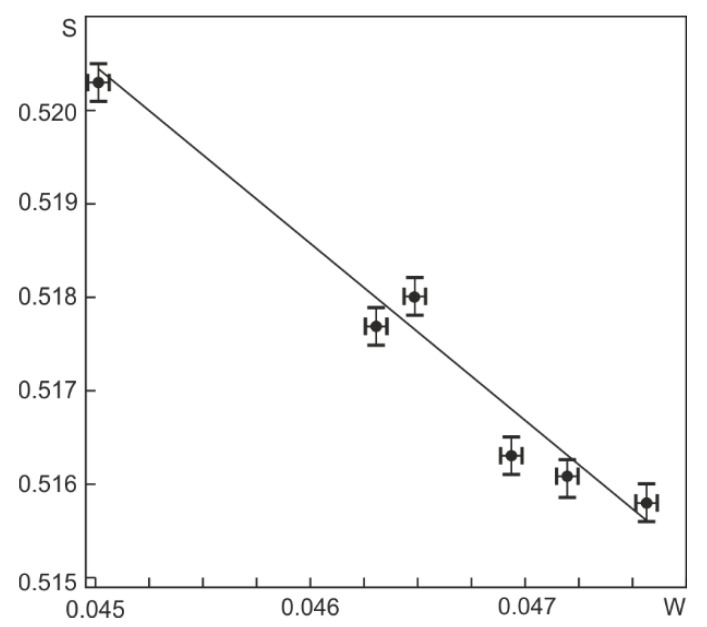
Dependence of S as a function of W for Ti_49.8_Ni_50.2_ samples after *abc* pressing with different values of the specified true strain.

The results obtained from the analysis of the positron lifetime spectra are confirmed by the data obtained by the DBS method. Figure 5 shows the dependence of the parameter S on the parameter W of the DBS spectrum. It can be seen that this dependence is linear. According to [37], this indicates that both in the initial samples and in the samples after *abc* pressing, the trap and annihilation of positrons are predominantly due to one type of defect in the crystal structure, which, as shown above, are dislocations.

**Figure 6:**
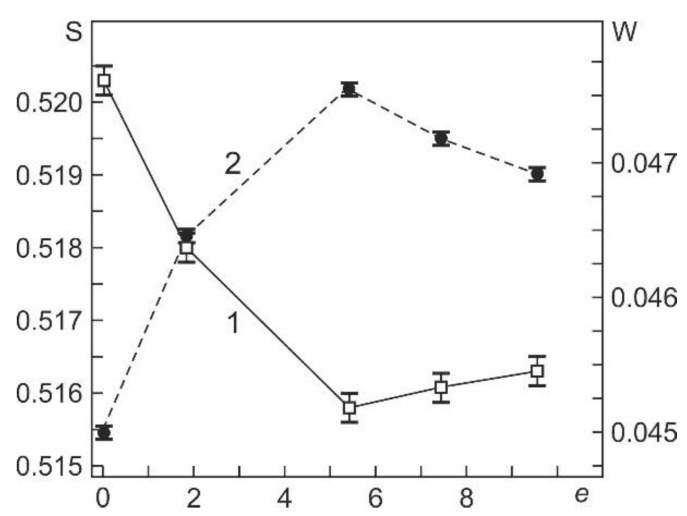
Dependences of DBS parameters S (1) and W (2) on the specified true strain *e* during *abc* pressing of the Ti_49.8_Ni_50.2_ alloy.

Figure 6 shows the DBS parameters S and W depending on the true deformation specified during *abc* pressing. It can be seen that the parameter S decreases with increasing *e* to 5.4, and after pressing with *e* from 5.4 to 9.55, it does not change within the error. The parameter W increases with increasing *e* to 5.4, and after pressing with *e* from 5.4 to 9.55, it tends to decrease. At the same time, these changes in the parameters S and W are not associated with a change in the concentration and type of defects, which are positron annihilation centers, since the positron lifetime τ_B_ and intensity I_B_ do not change (Table 2), and the dislocation density in the range *e* from 5.4 to 9.55 increases, as shown in Figure 4. Apparently, such dependences of S and W on *e* are due to the evolution of the dislocation structure of the alloy during pressing.

**Figure 7:**
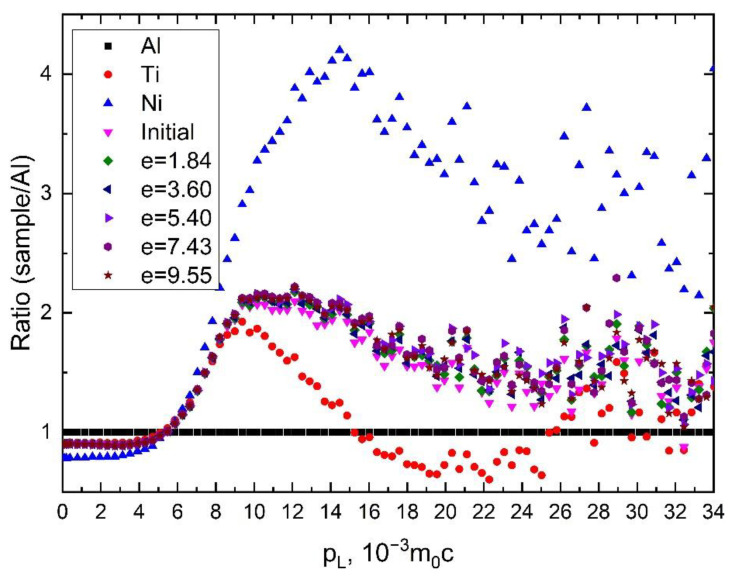
DBS measurements for samples of Ti, Ni and Ti_49.8_Ni_50.2_ alloy with different values of specified true strain *e* during *abc* pressing given as the ratio to that of annealed aluminum. The true strains are shown in insertion.

Figure 7 shows the dependences of the momentum distribution of positron annihilation for samples of pure Ti and Ni metals and the Ti_49.8_Ni_50.2_ alloy after pressing with an increasing value of the specified true strain *e*. Figure 7 shows that the ratio curves for the momentum distribution of positron annihilation for all samples of the Ti_49.8_Ni_50.2_ alloy is shifted toward the momentum distribution of positron annihilation in pure titanium. This shift is most pronounced for the initial samples, which contain defects in the form of vacancies in the nickel sublattice. Obviously, the regions in which positron annihilation occurs with lifetime τ_B_ are enriched in titanium. An increase in the true strain specified during *abc* pressing, when the τ_B_ component of the lifetime spectra is absent, leads to a slight shift in the positron annihilation momentum distribution toward a similar distribution for pure nickel. However, even in samples after *abc* pressing, positron annihilation occurs predominantly in microvolumes with a noticeable enrichment in titanium atoms. A more detailed discussion of this is given in the next section of the work.

## 4. Discussion

Using X-ray diffraction analysis, it was found that the value of the scalar dislocation density in the studied samples changes most significantly after *e* = 1.84: the dislocation density increases by almost an order of magnitude compared to the initial state of the samples (from 0.1 × 10^15^ m^−2^ to 1 × 10^15^ m^−2^). A subsequent increase in the *abc* strain to *e* = 9.55 leads to an increase in the dislocation density only by a factor of 2. From our point of view, this means that a significant part of the dislocations which appear during *abc* pressing take part in the formation of grain–subgrain boundaries. Due to the saturated positron trapping, the dislocation density and concentration of monovacancies cannot be determined exactly using the standard positron trapping model. However, it is possible to estimate the concentration of vacancies from the relation I_A_/I_B_ = ν_d_ρ_d_/ν_v_c_v_, based on positron trapping rate by dislocations ν_d_ and vacancies ν_v_ in metals, (≈10^−4^ m^2^s^−1^ and ≈10^14^ s^−1^, respectively) [66]. According to the X-ray diffraction data, the dislocation density ρ_d_ in the initial samples was ρ_d_ = 1.1 × 10^15^ m^−2^, as shown in Table 2. In this case, the evaluation shows that the relative concentration of V_Ni_ vacancies is ≈2 × 10^−5^, which exceeds their thermodynamically equilibrium concentration. Of course, as noted above, this is only an estimation, since the exact values of ν_d_ and ν_v_ for TiNi-based alloys are unknown. 

High concentrations of vacancies were obtained earlier. In particular, in nickel and copper after torsion under high pressure: 2 × 10^−3^ in Cu (ρ_d_ = 7 × 10^15^ m^−2^) [66] and 10^−2^ (ρ_d_ = 6.3 × 10^15^ m^−2^) in Ni [67]. After ECAP at room temperature of Cu, the concentration of vacancies was 4 × 10^−4^ (ρ_d_ = 2 × 10^15^ m^−2^) [68]. After ECAP at 723 K of the Ti_49.4_Ni_50.6_ (at.%) alloy, the concentration of vacancies is ≈10^−4^ (ρ_d_ = 10^15^ m^−2^) [63]. However, both in Cu after high-pressure torsion and in the Ti_49.4_Ni_50.6_ alloy after ECAP at 723 K, the lifetime spectra contain components due to positron annihilation both at dislocations and vacancies. 

Surprisingly, the high monovacancies concentration is observed in the initial samples of Ti_49.4_Ni_50.6_ alloy. At the same time, regardless of the value of *abc* strain, no free monovacancies are observed. In these samples, the trapping and annihilation of positrons are carried out by a single type of defects (dislocations). The physical reason for this effect remains unclear. The fact that we do not observe a positron lifetime component in vacancies can be due to the following reasons. This may be due to the fact that, with a decrease in the average grain–subgrain size, vacancies can migrate to sinks during deformation. The sinks for vacancies can be the increasing length of high-angle and low-angle grain boundaries and dislocations. In this regard, the recently published work [64] deserves special attention. In [64], the formation of defects in the crystal structure in samples of the Ti_49.45_Ni_50.55_ (at %) alloy after long-term thermal cycling through the temperature intervals of martensitic transformations under cooling and heating was studied by PALS. It is known [69,70,71,72,73] that such thermal cycling of samples of some binary TiNi-based alloys is accompanied by plastic deformation and, consequently, the appearance of a significant concentration of dislocations. The authors of [64] came to the conclusion that thermal cycling generates a type of defects in the form of dislocations with associated vacancies. The energy of vacancy formation in dislocations is less than the energy of formation of monovacancies in the bulk of the material [74]. The positron lifetime for dislocations with associated vacancies in thermally cycled samples of the Ti_49.45_Ni_50.55_ alloy is 160 ps [64]. This may be the basis for supposition that the vacancies formed during *abc* pressing of the Ti_49.8_Ni_50.2_ alloy are localized in dislocations. As a result, only one intensive component with τ_A_ = 166 ps is observed in the positron lifetime spectra for samples after *abc* pressing.

## 5. Conclusions

It is shown that samples of the Ti_49.8_Ni_50.2_ alloy, both in the initial state and after *abc* pressing at 573 K, have a two-level hierarchical microstructure. In the composition of large grains, the average size of which decreases from 27 µm in the initial state to 12 µm after pressing with *e* = 9.55, a submicrocrystalline grain–subgrain structure is formed (the average size of these grains–subgrains decreases from 0.36 µm in the initial state to 0.13 µm after pressing *e* = 9.55). The phase state of the samples at room temperature does not change after *abc* pressing at 573 K: rhombohedral R and monoclinic B19′ martensitic phases are present. In grains–subgrains, both monophase states (R or B19′) and two-phase states (R + B19′) are observed. 

X-ray diffraction analysis shows that the dislocation density increases by an order of magnitude (up to 10^15^ m^−2^) after *abc* pressing with *e* = 1.84 compared to the dislocation density in the initial samples (10^14^ m^−2^). The subsequent increase in the *abc* strain from 1.84 to 9.55 leads to an additional 2-fold increase in the dislocation density (up to 2 × 10^15^ m^−2^). 

It was established by positron annihilation lifetime spectroscopy that both in the initial samples and in the samples after *abc* pressing (regardless of the value of the specified true strain), there is a low-intensity (≤0.6%) component caused by positron annihilation from the delocalized state (positron lifetime 138 ± 1 ps), and saturated positron trapping is observed. The initial samples contain a long-lived component with a positron lifetime of 192 ± 2 ps, which corresponds to positron annihilation in monovacancies on the nickel sublattice of the Ti_49.8_Ni_50.2_ alloy, the intensity of which is 16.5%. This component is absent in the samples after *abc* pressing. The second component of the spectrum (positron lifetime 166 ± 1 ps), whose intensity increases from 83.4% in the initial samples to 99.4% in the samples after *abc* pressing, is due to the annihilation of positrons trapped by dislocations. The results of the analysis of the Doppler broadening spectroscopy confirm that: (i) the samples after *abc* pressing contain one type of defect in the crystal structure, which is an effective positron trapping center; (ii) the microvolumes in which positron annihilation occurs are enriched in titanium atoms. It is assumed that the absence of monovacancies in the samples after *abc* pressing is due either to their capture by dislocations or to the generation of dislocation type defects with associated vacancies directly during *abc* pressing.

## Figures and Tables

**Table 1:** Average value of grains–subgrains ⟨d⟩ depending on the value of the specified true strain *e* during *abc* pressing at 573 K.

*e*	0	1.84	3.60	5.40	7.43	9.55
⟨d⟩, μm	0.36	0.32	0.25	0.23	0.17	0.13

**Table 2:** Parameters of the components of the experimental positron annihilation lifetime spectra for the Ti_49.8_Ni_50.2_ alloy samples with different values of the specified true strain *e*: τ_F_, τ_A_, and τ_B_ are the positron lifetimes; I_A_ and I_B_ are the intensities of the A and B components, respectively ^1^.

Samples	τ_A_, ps	τ_B_, ps	τ_F_, ps	I_A_, %	I_B_, %
Initial	169 ± 1	192 ± 1	138 ± 1	83.4	16.54
*e* = 1.84	166 ± 1	-		99.9	-
*e* = 3.60	166 ± 1	-		99.5	-
*e* = 5.40	166 ± 1	-		99.6	-
*e* = 7.43	166 ± 1	-		99.7	-
*e* = 9.55	166 ± 1	-		99.4	-

^1^ Trapping rates k_A_ and k_B_ for the A and B types of defects are not presented in Table 2 because these data are not used for calculation of defect concentration according to the positron trapping model.

## Data Availability

Not applicable.
